# Molecular Mechanism Underlying IBRV-Induced Ferroptosis in MDBK Cells via the NFKB1-SLC39A8 Axis

**DOI:** 10.3390/ani16162580

**Published:** 2026-08-18

**Authors:** Yiming Wei, Wen Hao, Wanting Kou, Wenwen Yu, Xin Wang, Jianming Li, Guixue Hu, Kai Wang, Xue Leng

**Affiliations:** 1 College of Veterinary Medicine, Jilin Agricultural University, Changchun 130118, China; 18525182253@163.com (Y.W.); 18042618559@163.com (W.H.); 13384387290@163.com (W.K.); 16643010996@163.com (W.Y.); 15042172520@163.com (X.W.); huguixue901103@163.com (G.H.); wk197811@jlau.edu.cn (K.W.); 2 College of Chinese Medicine Materials, Jilin Agricultural University, Changchun 130118, China; lijianming773@jlau.edu.cn

**Keywords:** infectious bovine rhinotracheitis virus, ferroptosis, *SLC39A8*, *NFKB1*, molecular mechanism

## Abstract

Infectious bovine rhinotracheitis virus (IBRV) causes highly contagious bovine respiratory disease and threatens the global cattle industry. Ferroptosis, an iron-dependent regulated cell death, is involved in multiple viral infections, but its role and mechanism in IBRV infection remain unclear. This study investigated IBRV-induced ferroptosis and the *NFKB1-SLC39A8* axis in MDBK cells and examined the effect of ferroptosis on IBRV replication. Ferroptotic phenotypes and molecules were detected by biochemical assays, qPCR, Western blotting and TEM. Gain- and loss-of-function assays were performed to modulate gene expression, and their transcriptional relationship was verified by dual-luciferase assay. IBRV induced typical ferroptosis in MDBK cells, including Fe^2+^ overload, excessive ROS and MDA accumulation, GSH depletion and mitochondrial damage. Ferrostatin-1 reversed these changes and reduced viral titers, suggesting that ferroptosis contributes to IBRV replication. *SLC39A8* overexpression aggravated ferroptosis, whereas its knockdown suppressed it. Mechanistically, our data support that *NFKB1* positively regulates *SLC39A8* transcription, and *NFKB1* silencing inhibited IBRV-induced ferroptosis. In conclusion, this in vitro study shows that IBRV induces ferroptosis in MDBK cells via the *NFKB1-SLC39A8* axis, and ferroptosis may contribute to viral replication. These findings provide insights into IBRV–host cellular interaction and identify potential candidate molecular targets for future antiviral research.

## Simple Summary

Infectious bovine rhinotracheitis virus is a common pathogen that causes severe respiratory disease in cattle, resulting in heavy economic losses for cattle farmers worldwide. Current vaccines cannot fully eliminate latent viral infections in cattle, and there are no targeted therapeutic drugs available, so the disease remains hard to control effectively. This study aimed to investigate how the virus damages bovine cells and find potential targets for new prevention and treatment methods. We discovered that the virus triggers a special iron-related cell death process in bovine kidney cells: it leads to excess iron accumulation inside cells, generates harmful oxidative substances, and impairs mitochondria, the energy-generating organelles. Notably, ferroptosis represents one important pathogenic event during IBRV infection, alongside other well-documented cell death modalities. We also confirmed two key proteins that jointly regulate this cell death process. Inhibiting this iron-related cell death can effectively reduce viral replication. These findings reveal a new pathogenic mechanism of the virus, offer potential candidate targets and novel ideas for developing antiviral drugs, and will help protect cattle health and sustain the stable development of the cattle farming industry.

## 1. Introduction

Infectious Bovine Rhinotracheitis (IBR) is a severe contagious respiratory disease caused by Infectious Bovine Rhinotracheitis Virus (IBRV), also known as *Bovine herpesvirus 1* (BoHV-1) [1]. IBRV belongs to the Alphaherpesvirinae subfamily and can infect cattle of all ages, leading to respiratory distress, reproductive disorders, and even death in calves [2]. With the expansion of intensive cattle farming, the prevalence of IBR has shown an upward trend in many regions worldwide, causing significant economic losses to the global cattle industry [3]. Although vaccination is the main preventive measure, the latent infection characteristics of IBRV and the lack of specific therapeutic drugs make disease control challenging [4]. Therefore, clarifying the pathogenic mechanism of IBRV is crucial for developing novel control strategies.

Ferroptosis is a unique iron-dependent regulated cell death process characterized by excessive accumulation of lipid peroxides and mitochondrial damage, distinct from apoptosis, autophagy, and necrosis [5]. It is regulated by three core metabolic pathways: amino acid metabolism (the cystine/glutamate antiporter System Xc^−^/glutathione peroxidase 4 (GPX4) axis), iron metabolism, and lipid metabolism (acyl-CoA synthetase long-chain family member 4 (ACSL4)/polyunsaturated fatty acid (PUFA) axis) [6]. Iron overload-induced reactive oxygen species (ROS) production and lipid peroxidation are key drivers of ferroptosis [7]. Recent studies have shown that ferroptosis is involved in the pathogenesis of various viral infections, such as hepatitis B virus, influenza virus, and pseudorabies virus [8,9]. For example, pseudorabies virus induces ferroptosis by disrupting iron homeostasis to promote viral replication [10]. However, whether IBRV infection induces ferroptosis and the underlying molecular mechanism remain unknown.

*SLC39A8* (ZIP8), a member of the solute carrier family 39, is a divalent metal ion transporter that mediates the uptake of Fe^2+^, Zn^2+^, and Mn^2+^ [11]. *SLC39A8* plays a critical role in iron homeostasis and ferroptosis regulation. Accumulating evidence indicates that dysregulated SLC39A8 drives ferroptosis in multiple cell types via inducing intracellular iron overload and subsequent lipid peroxidation [12]. In inflammatory responses and viral infections, SLC39A8 expression is often upregulated, contributing to cell death and pathogenesis [13]. Nuclear Factor Kappa B1 (NFKB1) is a core transcription factor of the NF-κB signaling pathway, which participates in the modulation of immune responses, inflammation, and metal ion homeostasis [14]. NFKB1 can bind to specific response elements in the promoter region of target genes to regulate their transcription and is involved in the regulation of ferroptosis-related genes [15]. However, the regulatory relationship between *NFKB1* and *SLC39A8* in IBRV-induced ferroptosis has not been reported.

This study proposes the hypothesis that IBRV induces ferroptosis in MDBK cells via the *NFKB1-SLC39A8* signaling axis. To verify this hypothesis, we detected ferroptosis-related indicators in IBRV-infected cells, regulated the expression levels of *SLC39A8* and *NFKB1*, and further explored the regulatory effect of IBRV on ferroptosis through this signaling pathway on the basis of confirming the transcriptional regulatory relationship between *SLC39A8* and *NFKB1*. This study aims to reveal the novel pathogenic mechanism of IBRV at the cellular level and provide preliminary experimental reference and candidate targets for further antiviral research of IBRV.

## 2. Materials and Methods

### 2.1. Cells, Virus, and Reagents

Madin–Darby Bovine Kidney (MDBK) cells and Human Embryonic Kidney 293T (HEK-293T) cells were preserved in the Laboratory of Economic Animal Disease Prevention and Control, Jilin Agricultural University. The IBRV NM8 strain was isolated and identified by our laboratory. Dulbecco’s Modified Eagle Medium (DMEM), fetal bovine serum (FBS), and horse serum were purchased from Thermo Fisher Scientific (Waltham, MA, USA). Erastin, Ferrostatin-1 (Fer-1), and DMSO were obtained from MedChemExpress (Monmouth Junction, NJ, USA). Reactive Oxygen Species (ROS) Detection Kit, Malondialdehyde (MDA) Detection Kit, and Glutathione (GSH) Detection Kit were purchased from Beyotime Biotechnology (Shanghai, China). Ferrous Ion (Fe^2+^) Fluorescent Probe Kit was obtained from DOJINDO (Kumamoto, Japan). qPCR primers were synthesized by Sangon Biotech (Shanghai, China). Antibodies against SLC7A11 (no. BIO0928SM), GPX4 (no. DF6701), ACSL4 (no. DF12141), SLC39A8 (no. DF7743), FTH1 (no. DF6278), NFKB1 (no. BSM52309R), and GAPDH (no. BSM33033M) were purchased from Affinity Biosciences (Cincinnati, OH, USA) and Nature Biosciences (San Diego, CA, USA). Lentiviral vectors (pLenti-EF1a-EGFP-P2A-Puro-CMV-MCS-3Flag) and packaging plasmids (psPAX2, pMD2.G) were preserved in our laboratory. siRNAs targeting *SLC39A8* and *NFKB1* were synthesized by Genepharma (Shanghai, China). Dual-Luciferase Reporter Assay Kit was purchased from Promega (Madison, WI, USA).

### 2.2. Cell Culture and Virus Infection

MDBK cells and HEK-293T cells were cultured in DMEM supplemented with 10% FBS and 1% penicillin–streptomycin at 37 °C with 5% CO_2_. For virus infection, MDBK cells were seeded in culture plates and grown to 80% confluence, then inoculated with IBRV NM8 strain at a multiplicity of infection (MOI) of 0.01. This MOI was selected based on pre-experimental optimization: it induces stable and moderate cytopathic effect (CPE) at 36 h post-infection, avoids overwhelming non-specific necrosis caused by high-dose infection, and supports consistent detection of ferroptotic phenotypes. Infection efficiency was validated by monitoring CPE progression and viral titer determination via TCID_50_ assay, with approximately 70–80% of cells exhibiting typical CPE at 36 hpi. After incubation at 37 °C for 1 h, the virus solution was removed, and fresh DMEM medium containing 2% horse serum was added. The cells were cultured for 36 h before subsequent experiments. For all experiments, the uninfected control group was set as mock-infected MDBK cells: cells were incubated with an equal volume of maintenance medium (DMEM containing 2% horse serum) without virus for 1 h, and then cultured under the same conditions as infected groups. This mock-infection control was uniformly applied across all assays to ensure experimental consistency.

### 2.3. Drug Concentration Screening

Cell counting kit-8 (CCK-8) assay was used to screen the optimal concentrations of Erastin, Fer-1, and DMSO. MDBK cells were seeded in 96-well plates at a density of 1.5 × 10^4^ cells/well. After 24 h, the cells were treated with different concentrations of Erastin (1–12.5 μmol/L), Fer-1 (1–12.5 μmol/L), or DMSO (0.01–0.15%, *v*/*v*) for 36 h. CCK-8 solution was added to each well, and the absorbance at 450 nm was measured using a microplate reader. The optimal concentration was determined as the concentration with no significant cytotoxicity and effective biological activity.

### 2.4. Detection of Ferroptosis-Related Indicators

#### 2.4.1. Intracellular Fe^2+^ Detection

MDBK cells were seeded in laser confocal dishes at a density of 1 × 10^5^ cells/dish. After infection and drug treatment, the cells were stained with Hoechst 33342 for 20 min, then incubated with FerroOrange probe (1 μmol/L) for 30 min in the dark. The fluorescence intensity was observed using a laser confocal microscope (STELLARIS 5, Leica, Microsystems, Wetzlar, Germany). For quantitative analysis, at least 5 random non-overlapping fields were captured per sample, and the relative fluorescence intensity was analyzed using ImageJ software (version 1.53t; National Institutes of Health, Bethesda, MD, USA), with no fewer than 50 cells counted per field.

#### 2.4.2. ROS Detection

Cells were seeded in 6-well plates at a density of 3 × 10^5^ cells/well. After treatment, the cells were incubated with DCFH-DA probe (10 μmol/L) for 20 min in the dark. The cells were harvested, washed with PBS, and analyzed by flow cytometry (BD FACSVerse™, BD Biosciences, Franklin Lakes, NJ, USA).

#### 2.4.3. GSH and MDA Detection

Cells were harvested and lysed. GSH content was detected using GSH Detection Kit, and MDA level was measured using MDA Detection Kit according to the manufacturers’ instructions. Protein concentration was determined by bicinchoninic acid (BCA) assay to normalize the results.

#### 2.4.4. Mitochondrial Morphology Observation

Cells were fixed with 2.5% glutaraldehyde, dehydrated, embedded, and sectioned. The mitochondrial morphology was observed using a transmission electron microscope (HT-7800, Hitachi High-Technologies, Tokyo, Japan). For each experimental group, at least 10 random fields containing intact mitochondrial structures from 3 independent biological replicates were examined, with over 30 individual mitochondria evaluated per group. Representative images were selected for display.

### 2.5. qPCR and Western Blot Analysis

Total RNA was extracted using RNA Easy Fast Tissue/Cell Kit (Tiangen, Biotech, Beijing, China), and cDNA was synthesized using All-in-one First Strand cDNA Synthesis Kit II (Seven Biology, Beijing, China). qPCR was performed using TB Green Premix Ex Taq™ II (TaKaRa, Kusatsu, Shiga, Japan) with *GAPDH* as the internal reference gene. The primer sequences are listed in Table 1. The specificity of all primers was verified by melt curve analysis, with a single amplification peak observed for each gene. The amplification efficiency of all primer pairs was between 90% and 110%, which meets the standard for quantitative real-time PCR.

For Western blot, total proteins were extracted using RIPA lysis buffer (Yasase, Shanghai, China) containing protease and phosphatase inhibitors. Proteins were separated by sodium dodecyl sulfate polyacrylamide gel electrophoresis (SDS-PAGE), transferred to polyvinylidene fluoride (PVDF) membranes, and blocked with 5% skimmed milk in Tris-buffered saline with Tween 20 (TBST) for 1 h at room temperature. The membranes were then incubated with primary antibodies diluted in blocking solution overnight at 4 °C. The working dilution ratios of all primary antibodies were as follows: SLC7A11 (1:1000), GPX4/TFRC (1:1000), ACSL4/NOX4 (1:1000), SLC39A8/SLC40A1/SLC11A2 (1:1000), FTH1/FTL (1:1000), NFKB1 (1:1000), and GAPDH (1:5000). After washing with TBST, the membranes were incubated with horseradish peroxidase (HRP)-conjugated secondary antibodies (1:5000) for 2 h at room temperature. The bands were visualized using ECL chemiluminescence reagent (Tanon, Shanghai, China) and quantified using ImageJ software.

### 2.6. Construction of SLC39A8 Overexpressing Cell Line

The CDS sequence of bovine *SLC39A8* (NM_001205630.1) was inserted into the pLenti-EF1a-EGFP-P2A-Puro-CMV-MCS-3Flag vector. The recombinant plasmid (pLenti-SLC39A8) was verified by double enzyme digestion and sequencing. For lentivirus packaging, HEK-293T cells were co-transfected with pLenti-*SLC39A8* and packaging plasmids psPAX2 and pMD2.G at a mass ratio of 4:3:1 using PEI 40K reagent, at a DNA: polyethylenimine (PEI) mass ratio of 1:3. The lentivirus supernatant was collected at 48 h and 72 h post-transfection, filtered through a 0.45 μm filter, and stored at −80 °C. MDBK cells were infected with the lentivirus at a multiplicity of infection (MOI) of 10 with 5 μg/mL Polybrene supplementation. Stable *SLC39A8*-overexpressing cell lines (OE-*SLC39A8*-MDBK) were selected with 4 μg/mL puromycin for 7 consecutive days, with fresh medium replaced every 48 h. The overexpression efficiency was confirmed by qPCR and Western blotting.

### 2.7. siRNA Interference

MDBK cells were seeded in 6-well plates at a density of 2 × 10^5^ cells/well. After 24 h of culture, cells were transfected with siRNAs targeting *SLC39A8* or *NFKB1*, as well as non-targeting negative control siRNA, using siRNA-mate plus transfection reagent (Genepharma, Shanghai, China) according to the manufacturer’s protocol. The final working concentration of each siRNA was 50 nM in the culture medium, and the dosage ratio of siRNA-mate plus transfection reagent to siRNA was 5 μL per 100 pmol siRNA for each well of 6-well plates. For viral infection assays, cells were inoculated with IBRV at an MOI of 0.01 at 24 h after siRNA transfection. The interference efficiency was detected by qPCR and Western blot at 48 h post-transfection. All siRNA transfection experiments were performed in three independent biological replicates. The siRNA sequences are listed in Table 2.

### 2.8. Dual-Luciferase Reporter Assay

The promoter region of bovine *SLC39A8* was cloned into the pGL4-Basic vector to construct the reporter plasmid (pGL4-*SLC39A8*-pro). HEK-293T cells were seeded in 12-well plates and co-transfected with pGL4-*SLC39A8*-pro, pcDNA3.1-*NFKB1* (or pcDNA3.1 empty vector), and pRL-TK (internal reference plasmid) using PEI 40K transfection reagent. After 48 h, the cells were lysed, and the luciferase activity was detected using Dual-Luciferase Reporter Assay Kit. The relative luciferase activity was calculated as the ratio of firefly luciferase activity to Renilla luciferase activity.

### 2.9. Viral Titer Determination (TCID_50_ Assay)

MDBK cells were seeded in 96-well plates at a density of 1.5 × 10^4^ cells/well. After 24 h, the virus solution was serially diluted from 10^−1^ to 10^−10^, and 100 μL of each dilution was added to the wells. The cells were cultured at 37 °C with 5% CO_2_ for 72 h, and the cytopathic effect (CPE) was observed. The viral titer (TCID_50_/mL) was calculated using the Reed–Muench method.

### 2.10. Statistical Analysis

All data were analyzed using GraphPad Prism 9.0 software. All experiments were performed with three independent biological replicates (*n* = 3), defined as experiments conducted on separate days with independently prepared cell batches and virus stocks. Biochemical assays, qPCR and TCID_50_ assays included 3 technical replicates per sample to ensure detection reliability, and all statistical analyses were based on biological replicates.

Prior to statistical comparison, the Shapiro–Wilk test was used to assess the normality of data distribution, and Levene’s test was used to verify homogeneity of variance. All datasets in this study satisfied the assumptions of normality and equal variance, so parametric tests were applied. Differences between groups were compared using one-way analysis of variance (ANOVA) followed by Tukey’s multiple comparison test. Exact *p* values are reported in the text for key comparisons; for graphical presentation, significance levels are denoted as follows: *p* < 0.05 was considered statistically significant (* *p* < 0.05, ** *p* < 0.01, *** *p* < 0.001, **** *p* < 0.0001). Data are presented as the mean ± standard deviation (SD) from three independent experiments. All Western blot quantifications were derived from three independent biological replicates, and representative blot images are shown in the figures.

## 3. Result

### 3.1. IBRV Infection Induces Ferroptosis in MDBK Cells

Ferroptosis is commonly identified by a combination of biochemical, molecular and ultrastructural criteria, along with pharmacological rescue assays. In this study, we systematically characterized ferroptotic phenotypes from four core dimensions, intracellular iron homeostasis, redox and lipid peroxidation status, expression of ferroptosis-associated molecules, and mitochondrial morphological changes, combined with intervention using the specific ferroptosis inhibitor Ferrostatin-1.

#### 3.1.1. Optimal Drug Concentrations

**Figure 1:**
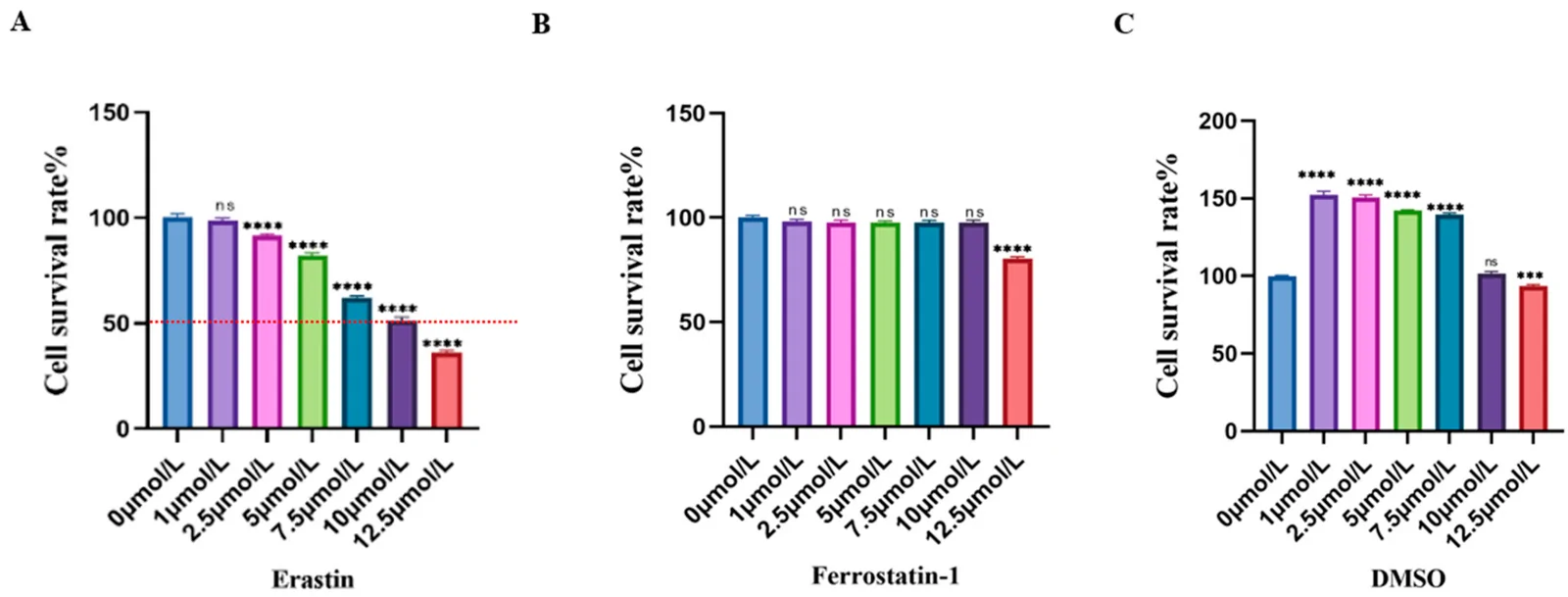
CCK-8 assay for optimal drug concentrations: (**A**) Cell viability after Erastin treatment. (**B**) Cell viability after Fer-1 treatment. (**C**) Cell viability after DMSO treatment. Cells were treated with drugs for 36 h before detection. Data are presented as mean ± SD from three independent biological replicates (*n* = 3). Statistical significance was analyzed by one-way ANOVA followed by Tukey’s multiple comparison test. For statistical significance analysis: *** indicates *p* < 0.001, **** indicates *p* < 0.0001, and ns indicates no significant difference.

To explore the optimal concentrations of the reagents used in this study, we analyzed the cytotoxicity of each reagent through the CCK-8 assay. Erastin, a classic ferroptosis inducer, was set as the positive control to verify the ferroptosis phenotype. The results showed that 10 μmol/L Erastin significantly reduced cell viability (*p* < 0.0001). Following the criterion of inducing measurable ferroptotic stress without excessive cytotoxicity, 10 μmol/L Erastin was selected as the working concentration for ferroptosis induction. Based on the criterion of no significant cytotoxicity and effective ferroptosis blockade, 10μmol/L Fer-1 was adopted as the ferroptosis inhibitor working concentration. 0.1% (*v*/*v*) DMSO was selected as the solvent control due to its minimal impact on cell viability (Figure 1).

#### 3.1.2. Ferroptosis-Related Biochemical Indicators

**Figure 2:**
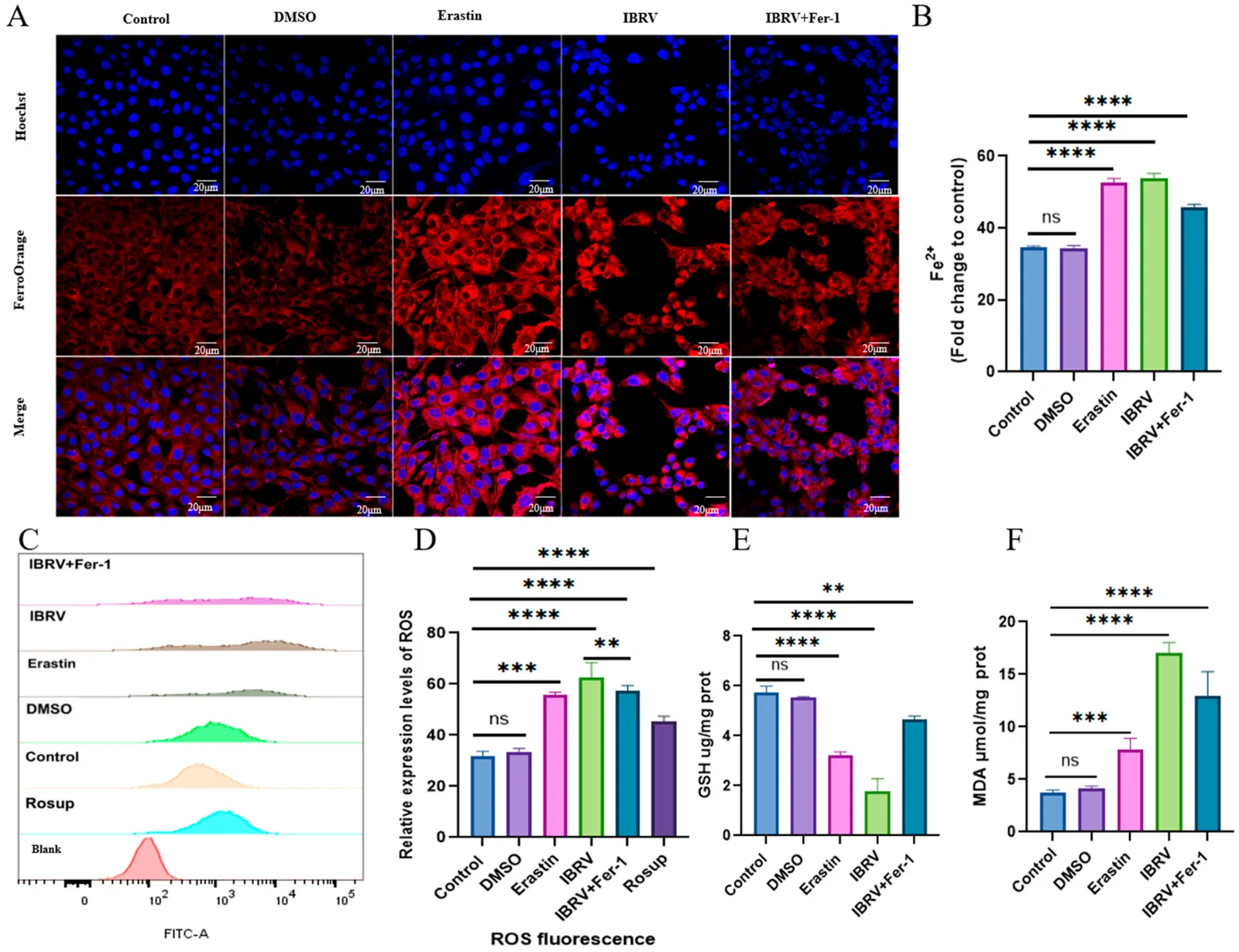
Detection of ferroptosis-related biochemical indicators: (**A**) Intracellular Fe^2+^ fluorescence intensity (400×). (**B**) Statistical analysis of Fe^2+^ relative fluorescence intensity. (**C**) ROS content detected by flow cytometry. (**D**) Statistical analysis of ROS relative fluorescence intensity. (**E**) GSH content; (**F**) MDA level. MDBK cells were infected with IBRV at an MOI of 0.01, and all samples were collected at 36 h post-infection (hpi). Data are presented as mean ± SD from three independent biological replicates (*n* = 3). For statistical significance analysis: ** indicates *p* < 0.01, *** indicates *p* < 0.001, **** indicates *p* < 0.0001, and ns indicates no significant difference.

To investigate the effects of IBRV infection on ferroptosis-related biochemical indicators in MDBK cells, this study detected ferroptosis-related biochemical indicators in infected cells using research methods such as laser confocal microscopy and flow cytometry. Compared with the control group, IBRV infection significantly increased intracellular Fe^2+^ fluorescence intensity (*p* < 0.0001), reactive oxygen species (ROS) content (*p* < 0.0001), and malondialdehyde (MDA) level (*p* < 0.0001) while significantly decreasing glutathione (GSH) content (*p* < 0.0001). Erastin treatment induced consistent changes across all four detected indicators. Fer-1 treatment significantly attenuated all these IBRV-induced alterations (Figure 2). These biochemical changes are consistent with the hallmark features of ferroptosis.

#### 3.1.3. Mitochondrial Morphological Changes

**Figure 3:**
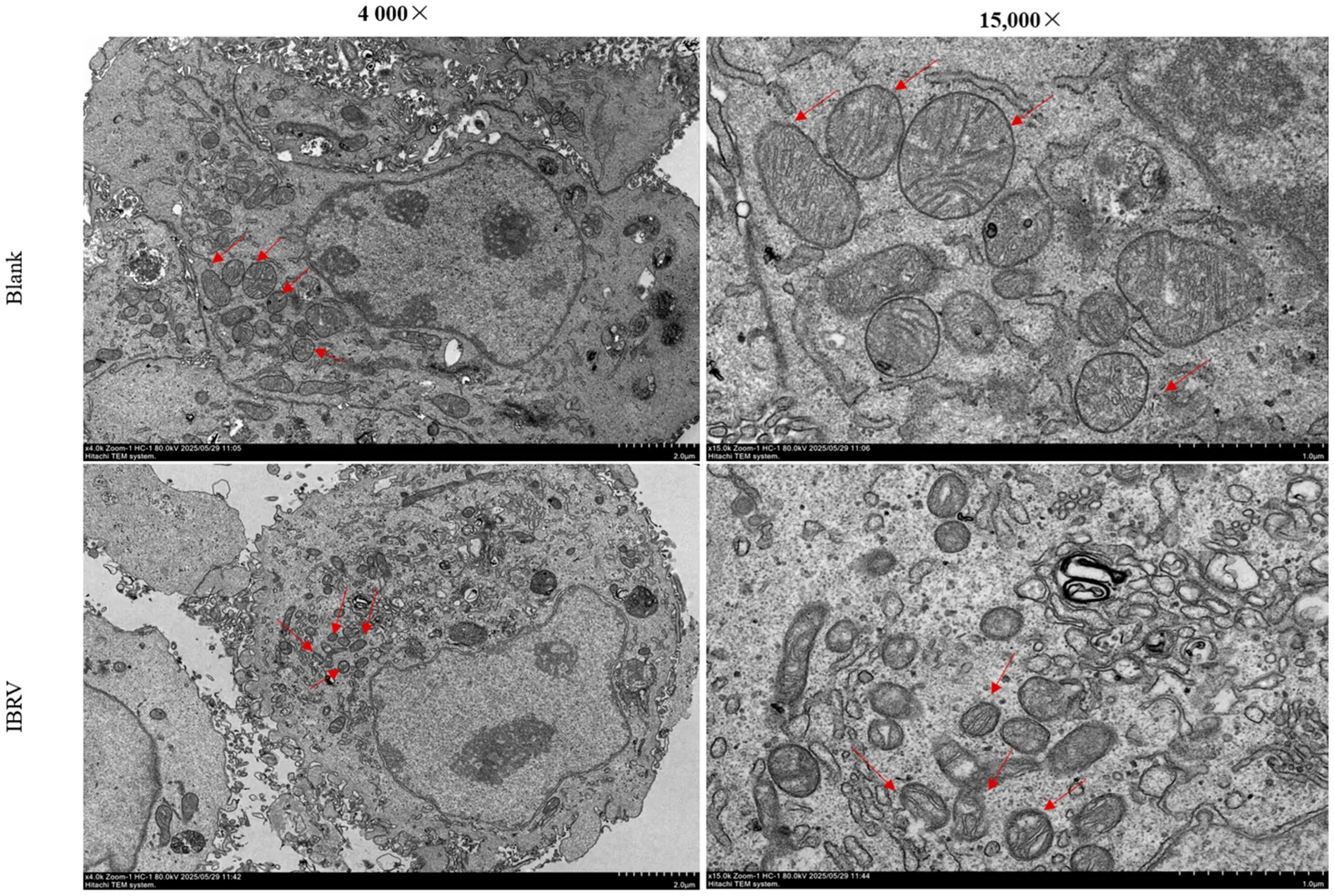
Mitochondrial morphological changes observed by transmission electron microscopy. Left panel: Scale bar = 2 μm; Right panel: Scale bar = 1 μm (enlarged view of the red arrow area in the left panel). Red arrows indicate mitochondria. MDBK cells were infected with IBRV at an MOI of 0.01 and samples were collected at 36 hpi. Images are representative of three independent biological experiments.

To investigate the effect of IBRV infection on mitochondrial morphology in MDBK cells, this study employed transmission electron microscopy (TEM) for observation, and the results showed that MDBK cells in the control group had intact mitochondria with clear cristae. In contrast, IBRV-infected cells exhibited mitochondrial cristae rupture, reduction or disappearance, and increased membrane density, which are consistent with the typical morphological characteristics of ferroptosis (Figure 3), This result indicates that IBRV infection induces typical ferroptotic morphological changes in the mitochondria of MDBK cells.

#### 3.1.4. Expression of Ferroptosis-Related Genes and Proteins

**Figure 4:**
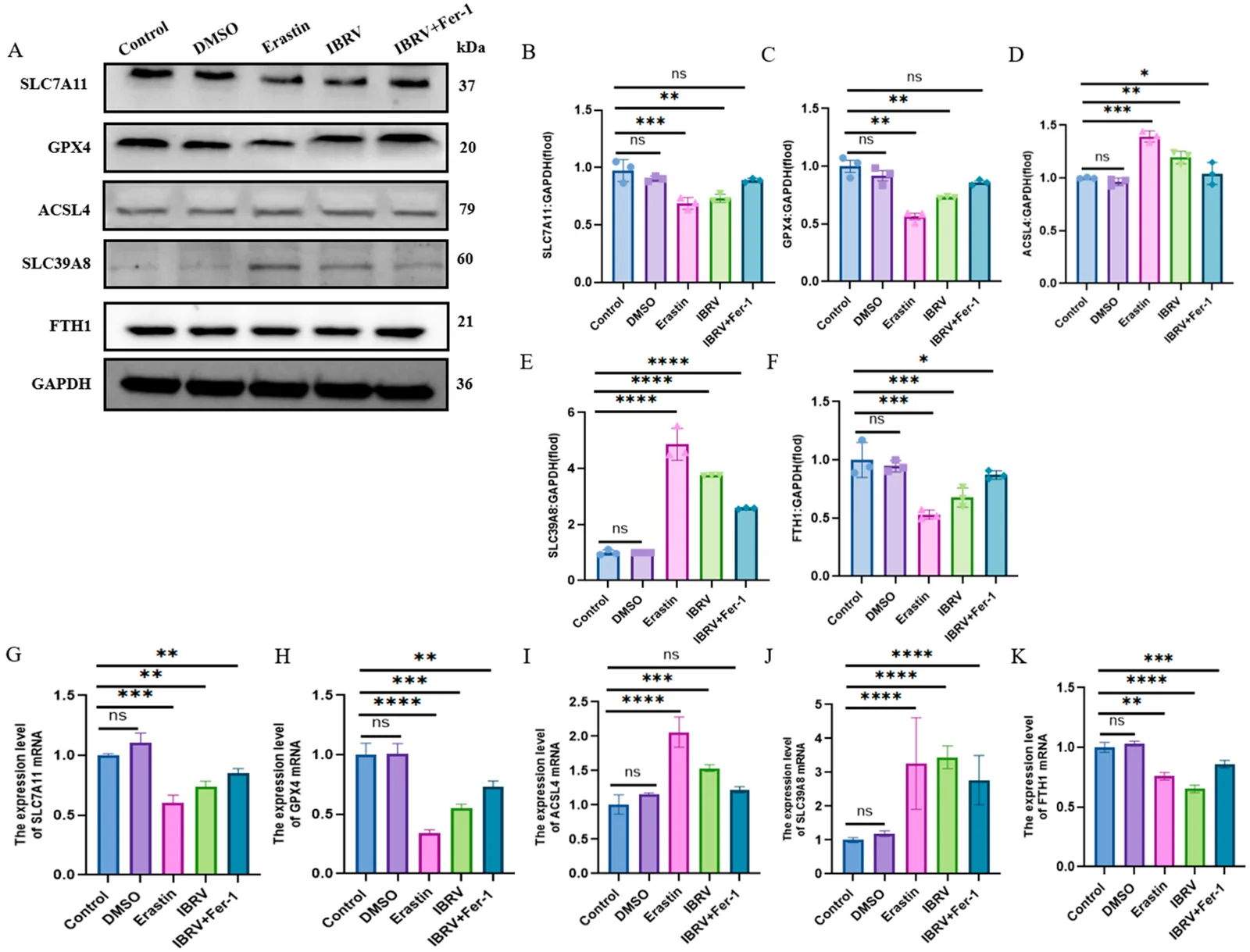
Expression of ferroptosis-related genes and proteins (**A**–**F**) Western blot results for SLC7A11, GPX4, ACSL4, and GAPDH; Western blot results for SLC39A8, FTH1, and GAPDH; statistical analysis of protein expression levels. (**G**–**K**) mRNA expression levels detected by qPCR. MDBK cells were infected with IBRV at an MOI of 0.01 and samples were collected at 36 hpi. Data are presented as mean ± SD from three independent biological replicates (*n* = 3). For statistical significance analysis: * indicates *p* < 0.05, ** indicates *p* < 0.01, *** indicates *p* < 0.001, **** indicates *p* < 0.0001, and ns indicates no significant difference.

To investigate the effects of IBRV infection on the expression of ferroptosis-related genes and proteins in MDBK cells, this study employed qPCR and Western blot detection, and the results showed that IBRV infection significantly downregulated the mRNA and protein expression levels of solute carrier family 7 member 11 (*SLC7A11*), *GPX4*, and ferritin heavy chain 1 (*FTH1*) (*p*< 0.01) while significantly upregulating the expression of *ACSL4* and *SLC39A8* (*p* < 0.01). Fer-1 treatment effectively reversed these expression changes (Figure 4). IBRV infection can induce changes in the expression levels of molecules associated with the ferroptosis pathway.

### 3.2. Pharmacological Inhibition of Ferroptosis Suppresses IBRV Replication

**Figure 5:**
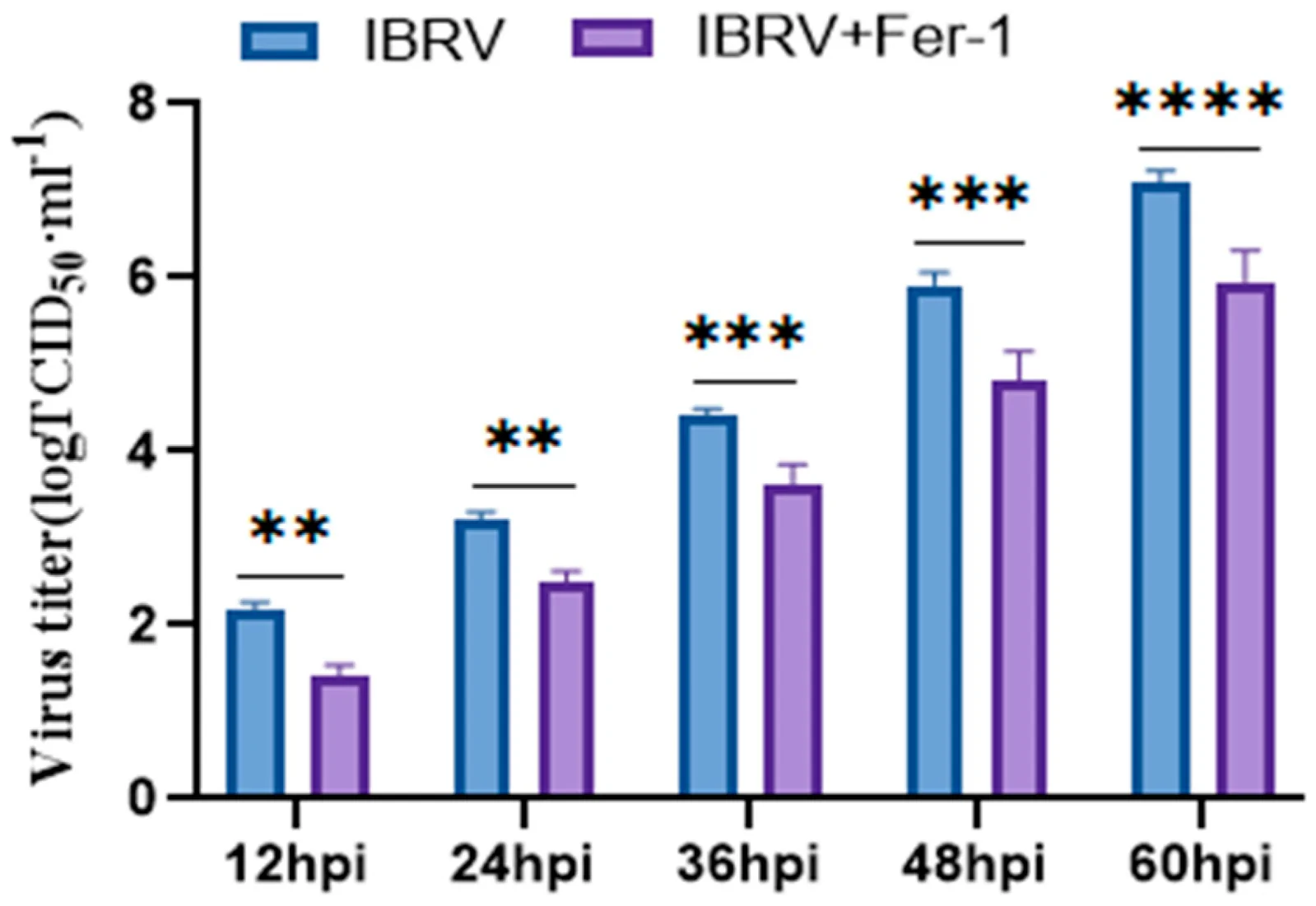
Effect of Fer−1 on IBRV replication detected by TCID_50_ assay. MDBK cells were infected with IBRV at an MOI of 0.01, and viral titers were determined at 24 hpi, 36 hpi, 48 hpi, and 60 hpi respectively. Data are presented as mean ± SD from three independent biological replicates (*n* = 3). For statistical significance analysis: ** indicates *p* < 0.01, *** indicates *p* < 0.001, **** indicates *p* < 0.0001.

To investigate the role of ferroptosis in the replication of IBRV, this study conducted detection via the TCID_50_ assay, and the results showed that compared with the IBRV infection group, Fer-1 treatment significantly reduced the viral titer at 24 hpi, 36 hpi, 48 hpi, and 60 hpi post-infection (*p* < 0.01). Pharmacological blockade of ferroptosis by Fer-1 significantly inhibits IBRV replication, which suggests a potential pro-viral effect of ferroptosis during IBRV infection (Figure 5).

### 3.3. SLC39A8 Promotes IBRV-Induced Ferroptosis

#### 3.3.1. Construction of SLC39A8 Overexpression Cell Line

**Figure 6:**
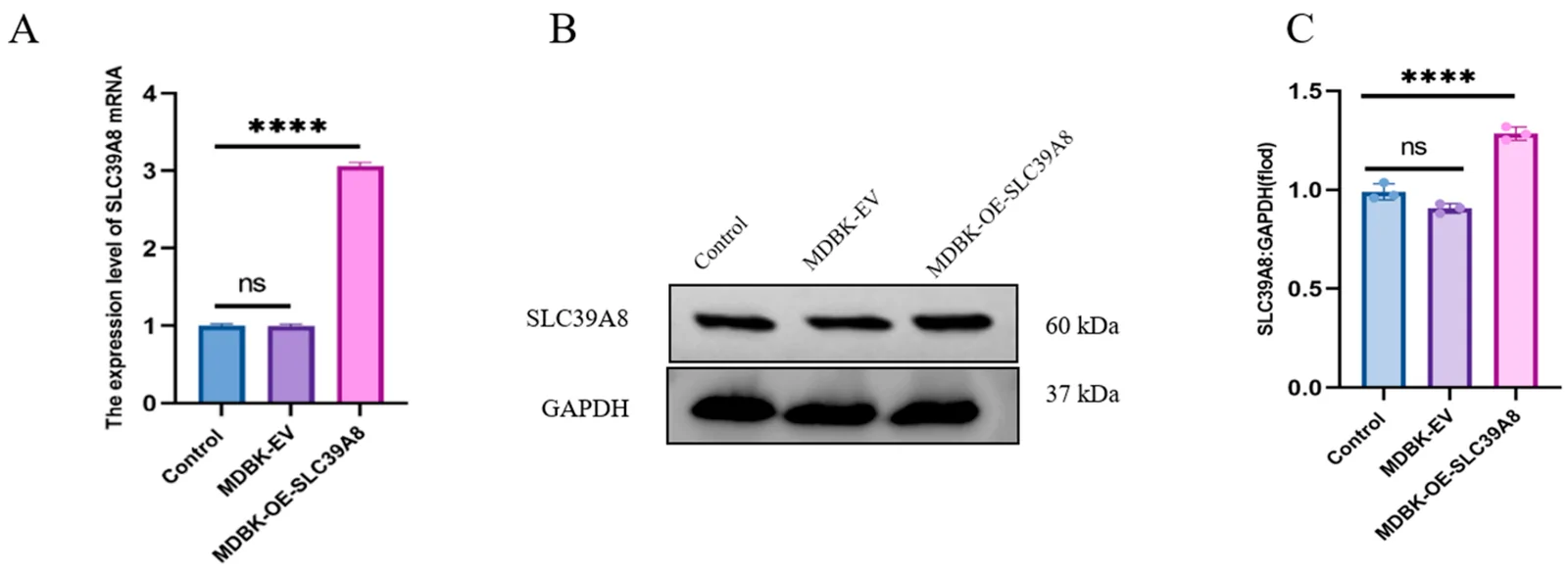
Verification of SLC39A8 overexpression cell line: (**A**) qPCR detection of *SLC39A8* mRNA expression. (**B**) Western blot detection of SLC39A8 protein expression. (**C**) Statistical analysis of protein expression levels. Assays were performed in stable puromycin-selected MDBK cells. Data are presented as mean ± SD from three independent biological replicates (*n* = 3). For statistical significance analysis: **** indicates *p* < 0.0001, and ns indicates no significant difference.

To verify the successful construction of the *SLC39A8* overexpression cell line, this study employed double enzyme digestion and DNA sequencing for validation, the results showed that pLenti-*SLC39A8* vector was constructed successfully (Supplementary Figure S1). The titer of the lentivirus was 3.1 × 10^6^ TU/mL. qPCR and Western blot results showed that the mRNA and protein expression levels of SLC39A8 in OE-*SLC39A8*-MDBK cells were significantly higher than those in the control group (*p* < 0.0001), confirming the successful construction of the *SLC39A8* overexpression cell line (Figure 6). 

#### 3.3.2. Effect of SLC39A8 Overexpression on Ferroptosis

**Figure 7:**
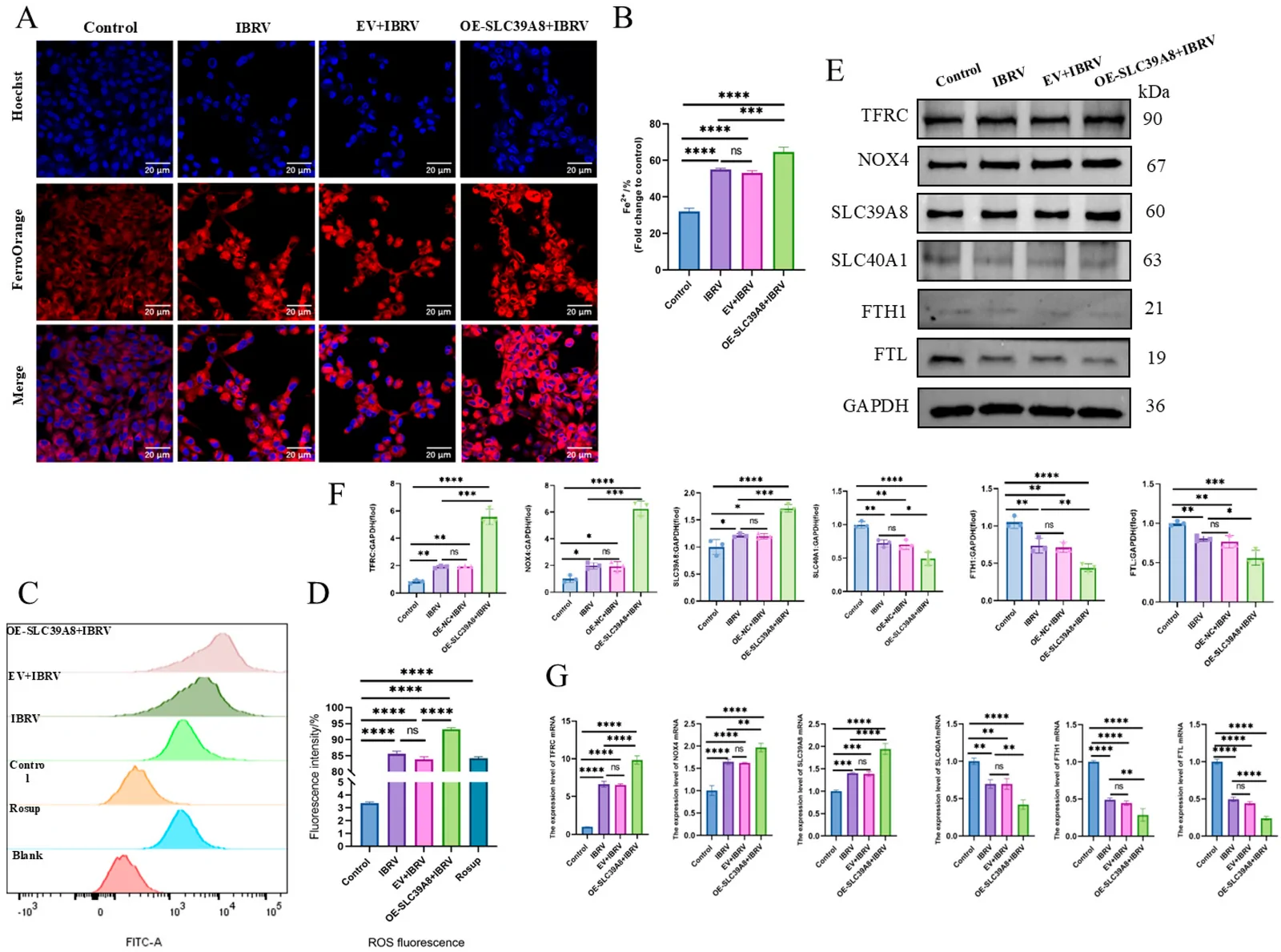
Effect of *SLC39A8* overexpression on IBRV−induced ferroptosis: (**A**) Intracellular Fe^2+^ fluorescence intensity (400×). Note: The scale bar in the figure is 20 μm. (**B**) Statistical analysis of Fe^2+^ relative fluorescence intensity. (**C**) ROS content detected by flow cytometry. (**D**) Statistical analysis of ROS relative fluorescence intensity; (**E**,**F**) Western blot results and statistical analysis of iron metabolism-related proteins. (**G**) mRNA expression levels detected by qPCR. Stable *SLC39A8*−overexpressing MDBK cells were infected with IBRV at an MOI of 0.01 and samples were collected at 36 hpi. Data are presented as mean ± SD from three independent biological replicates (*n* = 3). For statistical significance analysis: * indicates *p* < 0.05, ** indicates *p* < 0.01, *** indicates *p* < 0.001, **** indicates *p* < 0.0001, and ns indicates no significant difference.

To investigate the effect of *SLC39A8* gene overexpression on ferroptosis-related biochemical indicators in infected cells, this study detected ferroptosis-related biochemical indicators after cell treatment using research methods including laser confocal microscopy and flow cytometry. The results showed that compared with the IBRV infection group, OE-*SLC39A8*-MDBK cells infected with IBRV exhibited significantly increased intracellular Fe^2+^ content (*p* < 0.0001) and ROS level (*p* < 0.0001). The expression levels of *TFRC*, *NOX4*, and *SLC39A8* were significantly upregulated (*p* < 0.001), while the expression levels of *SLC40A1*, *FTH1*, and *FTL* were significantly downregulated (*p* < 0.05) (Figure 7). Overexpression of SLC39A8 promotes the increase in intracellular Fe^2+^ content in infected cells, induces the excessive production of ROS, and accelerates the ferroptosis process induced by IBRV. 

#### 3.3.3. Effect of SLC39A8 Silencing on Ferroptosis

**Figure 8:**
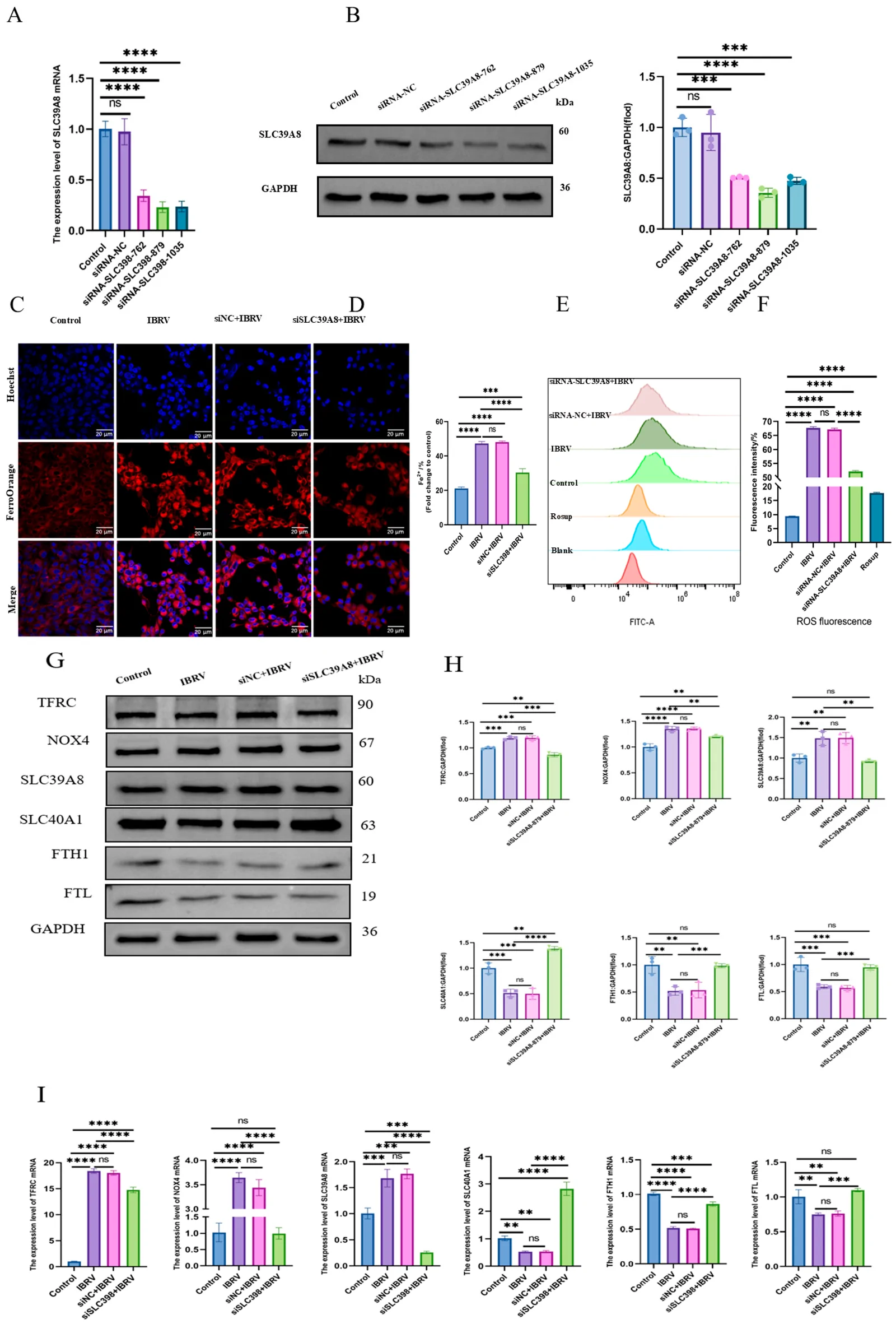
Effect of *SLC39A8* silencing on IBRV-induced ferroptosis: (**A**) Interference efficiency of siRNA-*SLC39A8* detected by qPCR. (**B**) Interference efficiency detected by Western blot. (**C**) Intracellular Fe^2+^ fluorescence intensity (400×). Note: The scale bar in the figure is 20 μm. (**D**) Statistical analysis of Fe^2+^ relative fluorescence intensity. (**E**) ROS content detected by flow cytometry. (**F**) Statistical analysis of ROS relative fluorescence intensity. (**G**–**I**) Western blot and qPCR results of iron metabolism-related genes and proteins. MDBK cells were transfected with siRNA for 24 h, then infected with IBRV at an MOI of 0.01, and samples were collected at 36 hpi. Data are presented as mean ± SD from three independent biological replicates (*n* = 3). For statistical significance analysis: ** indicates *p* < 0.01, *** indicates *p* < 0.001, **** indicates *p* < 0.0001, and ns indicates no significant difference.

To investigate the effect of *SLC39A8* gene silencing on ferroptosis-related biochemical indicators in infected cells, this study detected ferroptosis-related biochemical indicators after cell treatment using research methods including laser confocal microscopy and flow cytometry. The results showed that siRNA-*SLC39A8*-879 showed the highest interference efficiency (*p* < 0.0001). Compared with the IBRV infection group, *SLC39A8*-silenced cells showed significant decrease in intracellular Fe^2+^ content (*p* < 0.0001) and ROS level (*p* < 0.0001). The expression level of *TFRC*, *NOX4*, and *SLC39A8* were significantly downregulated (*p* < 0.01), while the expression levels of *SLC40A1*, *FTH1*, and *FTL* were significantly upregulated (*p* < 0.001) (Figure 8). The successful silencing of the *SLC39A8* gene. Interfering with the expression of the *SLC39A8* gene inhibited the increase in Fe^2+^ content and ROS production in infected cells and suppressed the ferroptosis process induced by IBRV.

### 3.4. NFKB1 Regulates SLC39A8 Transcription to Mediate IBRV-Induced Ferroptosis

#### 3.4.1. NFKB1 Activates SLC39A8 Transcription

**Figure 9:**
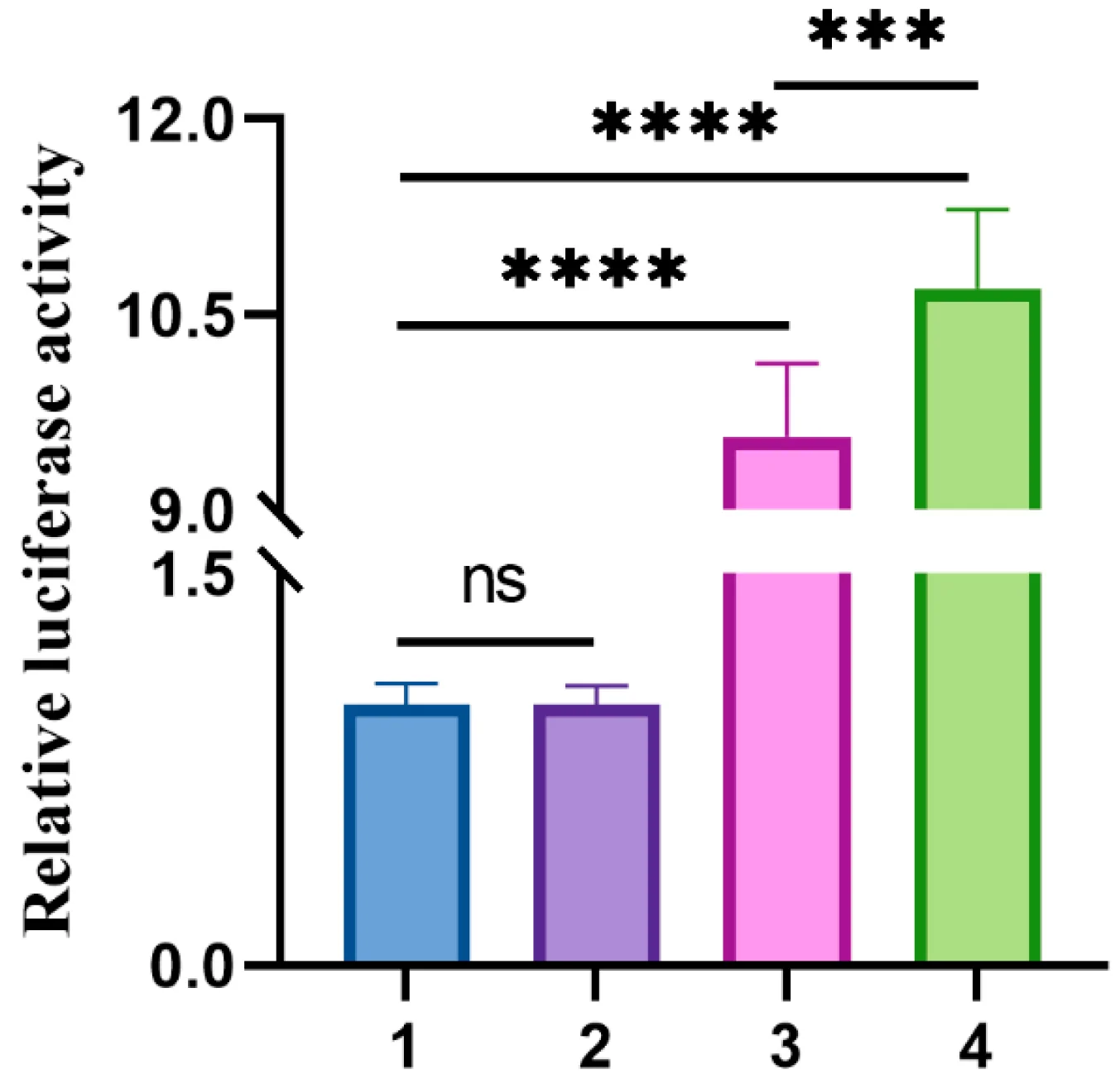
Effect of NFKB1 on the transcriptional activity of the *SLC39A8* promoter detected by dual-luciferase reporter assay. 1: pGL4-Basic + pcDNA3.1 + pRL-TK; 2: pGL4-Basic + pcDNA3.1-*NFKB1* + pRL-TK; 3: pGL4-*SLC39A8*-pro + pcDNA3.1 + pRL-TK; 4: pGL4-*SLC39A8*-pro + pcDNA3.1-*NFKB1* + pRL-TK. HEK-293T cells were co-transfected with the indicated plasmids, and luciferase activity was detected at 48 h post-transfection. Data are presented as mean ± SD from three independent biological replicates (*n* = 3). For statistical significance analysis: *** indicates *p* < 0.001, **** indicates *p* < 0.0001, and ns indicates no significant difference.

To investigate the effect of NFKB1 on the *SLC39A8* in transcription, dual-luciferase reporter assay results showed that compared with the control group, co-transfection of pcDNA3.1-*NFKB1* and pGL4-*SLC39A8*-pro significantly increased the relative luciferase activity (*p* < 0.0001). These results suggest that *NFKB1* exerts a transcriptional activation effect on the *SLC39A8* promoter (Figure 9).

#### 3.4.2. Effect of NFKB1 Silencing on Ferroptosis

**Figure 10:**
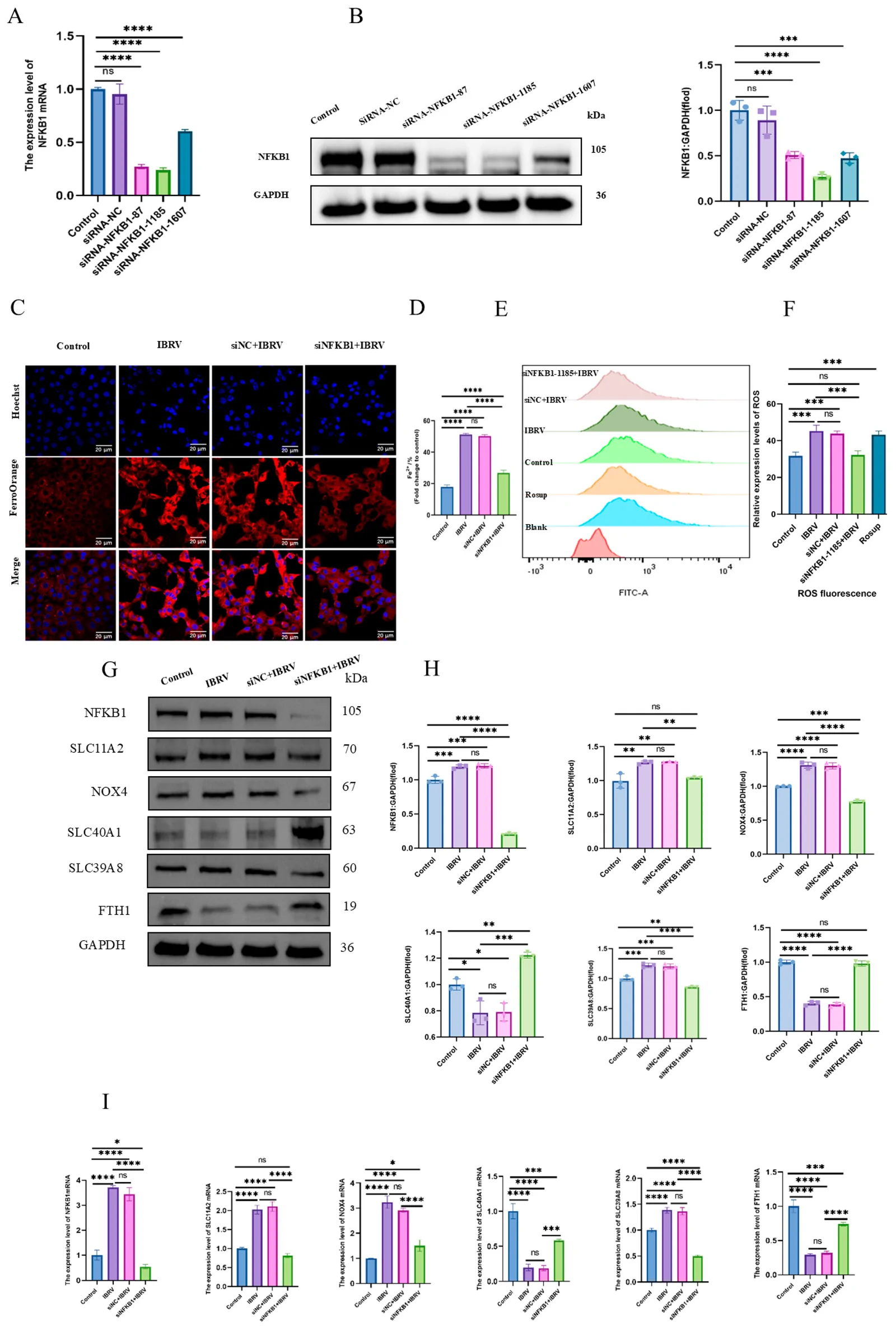
Effect of *NFKB1* silencing on IBRV-induced ferroptosis: (**A**) Interference efficiency of siRNA−*NFKB1* detected by qPCR. (**B**) Interference efficiency detected by Western blot. (**C**) Intracellular Fe^2+^ fluorescence intensity (400×). Note: The scale bar in the figure is 20 μm. (**D**) Statistical analysis of Fe^2+^ relative fluorescence intensity. (**E**) ROS content detected by flow cytometry. (**F**) Statistical analysis of ROS relative fluorescence intensity. (**G**−**I**) Western blot and qPCR results of iron metabolism−related genes and proteins. MDBK cells were transfected with siRNA for 24 h, then infected with IBRV at an MOI of 0.01, and samples were collected at 36 hpi. Data are presented as mean ± SD from three independent biological replicates (*n* = 3). For statistical significance analysis: * indicates *p* < 0.05, ** indicates *p* < 0.01, *** indicates *p* < 0.001, **** indicates *p* < 0.0001, and ns indicates no significant difference.

To investigate the effect of *NFKB1* gene silencing on ferroptosis-related biochemical indicators in infected cells, this study detected ferroptosis-related biochemical indicators after cell treatment using research methods including laser confocal microscopy and flow cytometry. The results showed that siRNA-*NFKB1*-1185 showed the highest interference efficiency (*p* < 0.0001). Compared with the IBRV infection group, *NFKB1*-silenced cells showed significant decrease in intracellular Fe^2+^ content (*p* < 0.0001) and ROS level (*p* < 0.001). The expression levels of *NFKB1*, solute carrier family 11 member 2 (*SLC11A2*), *NOX4*, and *SLC39A8* were significantly downregulated (*p* < 0.01), while the expression levels of *SLC40A1* and *FTH1* were significantly upregulated (*p* < 0.001) (Figure 10). These results indicate that siRNA-*NFKB1* was successfully constructed, and interfering with *NFKB1* expression significantly reduced intracellular Fe^2+^ and ROS levels and suppressed IBRV-induced ferroptosis, suggesting that NFKB1 is required for this process.

## 4. Discussion

Ferroptosis, a novel iron-dependent regulated cell death distinct from apoptosis, necrosis, and autophagy, is characterized by iron-dependent lethal accumulation of lipid peroxides and has become a research hotspot in the field of viral pathogenesis in recent years [16]. Accumulating evidence indicates that numerous viruses, including herpesviruses, influenza viruses, and coronaviruses, can manipulate host ferroptosis to facilitate their own replication and pathogenicity, highlighting the close association between ferroptosis dysregulation and viral infection outcomes [9,17]. As far as we know, this study is the first to report that IBRV infection induces ferroptosis in MDBK cells, and further clarifies the regulatory role of the *NFKB1-SLC39A8* axis in this process. Our findings show that IBRV infection disrupts host iron metabolism homeostasis, triggers iron overload, excessive ROS accumulation and lipid peroxidation, and induces ferroptosis-like phenotypes, which may contribute to a cellular state supportive of IBRV replication.

Iron overload is a key initiator of ferroptosis, and the precise regulation of iron metabolism (including iron uptake, storage, release, and export) is critical for maintaining ferroptosis homeostasis [18]. In this study, our results showed that IBRV infection significantly increased intracellular Fe^2+^ content in MDBK cells, which was closely associated with the dysregulated expression of iron metabolism-related molecules. Specifically, IBRV infection significantly upregulated the expression of iron uptake transporters (*SLC39A8* and *TFRC*) and downregulated the expression of iron storage proteins (*FTH1*, *FTL*) and iron export protein (*SLC40A1*), leading to abnormal iron influx and reduced iron efflux/storage, ultimately resulting in intracellular iron overload. Among these molecules, *SLC39A8* (ZIP8), a ZIP-family divalent metal transporter that primarily mediates cellular uptake of zinc and manganese, with ferrous iron as one of its transport substrates, was significantly upregulated after IBRV infection, and its regulatory role in IBRV-induced ferroptosis was further confirmed by gain-of-function and loss-of-function experiments: overexpression of *SLC39A8* further increased intracellular Fe^2+^ content, exacerbated ROS accumulation and lipid peroxidation, and promoted ferroptosis, while silencing SLC39A8 had the opposite effect, effectively inhibiting IBRV-induced ferroptosis. This finding is consistent with the latest research progress showing that SLC39A8 contributes to intracellular iron accumulation and participates in ferroptosis regulation in various cell types, including cancer cells and epithelial cells [12,19]. Notably, recent studies have also found that SLC39A8 can regulate ferroptosis by interacting with other iron metabolism-related molecules (such as TFRC and SLC40A1) to form a complex regulatory network, which may also be involved in IBRV-induced ferroptosis and deserves further exploration in subsequent studies [12].

ROS accumulation and lipid peroxidation are important biochemical characteristics of ferroptosis and the imbalance between oxidative stress and antioxidant defense systems is the core mechanism driving ferroptosis progression [20]. Our study showed that IBRV infection significantly increased intracellular ROS and MDA levels while significantly decreasing GSH content. GSH is an important antioxidant that maintains the activity of *GPX4*, a key lipid peroxidation inhibitor that can scavenge lipid peroxides and prevent ferroptosis [21]. Consistent with this, we found that IBRV infection significantly downregulated GPX4 protein expression, though its enzymatic activity was not directly measured in this study, which may have further impaired the antioxidant capacity of host cells and promoted lipid peroxidation. Importantly, treatment with Fer-1, a lipid radical scavenger that suppresses lipid peroxidation, significantly attenuated IBRV-induced alterations in Fe^2+^, ROS, GSH and MDA levels and significantly reduced viral titers, suggesting an association between ferroptosis and enhanced IBRV replication. Notably, Fer-1 may exert broad cytoprotective effects beyond ferroptosis inhibition due to its antioxidant properties. However, given that we detected a complete set of canonical ferroptotic hallmarks upon IBRV infection, including intracellular iron overload, GSH depletion, GPX4 downregulation and typical mitochondrial morphological damage, the reduction in viral titer may be largely attributed to ferroptosis blockade rather than non-specific antioxidant effects, though the contribution of Fer-1’s broad antioxidant activity cannot be fully excluded. This observation aligns with previous reports in pseudorabies virus (PRV) and influenza virus models, noting that the functional role of ferroptosis may vary across viral species, cell types and infection stages, where ferroptosis inhibition was shown to reduce viral replication [22,23]. From the perspective of viral infection, ferroptosis may benefit IBRV propagation by promoting cell lysis and progeny virus release, and shaping an oxidative microenvironment favorable for viral replication. Damage-associated molecular patterns (DAMPs) released during ferroptosis may further mediate local immunosuppression, which is potentially associated with the immune evasion mechanisms of BoHV-1 [24]. Meanwhile, the possibility that ferroptosis acts as a host defense response to eliminate infected cells and restrict viral spread cannot be fully ruled out. In addition, given the broad cytoprotective and antioxidant activities of Fer-1, its inhibitory effect on viral replication may also be partially mediated by improving host cell viability rather than exclusively by blocking ferroptosis. Notably, the potential mechanism by which ferroptosis facilitates viral replication via damage-associated molecular pattern (DAMP)-mediated inflammatory amplification and immunosuppression has been proposed in existing published studies but was not experimentally validated in the present work, as we did not measure DAMP levels, inflammatory responses, or host antiviral immunity [25]. This putative mechanism remains to be systematically verified in subsequent studies.

NFKB1, a core transcription factor of the NF-κB signaling pathway, is widely involved in the regulation of various cellular processes, including inflammation, cell proliferation, apoptosis, iron metabolism and ferroptosis [26]. In this study, combined evidence from the dual-luciferase reporter assay and *NFKB1* loss-of-function assays supports that NFKB1 transcriptionally regulates *SLC39A8* expression: in the dual-luciferase reporter assay performed in HEK-293T cells, overexpression of *NFKB1* enhanced the transcriptional activity of the *SLC39A8* promoter, while silencing *NFKB1* in MDBK cells significantly downregulated endogenous *SLC39A8* expression, reduced intracellular iron overload and ROS accumulation, and ultimately inhibited IBRV-induced ferroptosis. These results collectively suggest that the *NFKB1-SLC39A8* axis is involved in mediating IBRV-induced ferroptosis in MDBK cells. Combined with the finding that ferroptosis inhibition reduces viral replication, it is plausible that modulation of the *NFKB1-SLC39A8* axis may affect IBRV replication by regulating ferroptosis levels, although direct experimental validation is still lacking. Consistent with our findings, previous studies have shown that *NFKB1* can regulate the expression of ferroptosis-related genes such as *FDX1* and *SLC7A11* [27], suggesting that NFKB1 may act as a key transcription factor to regulate ferroptosis through multiple targets.

Notably, NF-κB signaling plays a dual role in viral infection: it functions as a core host regulator involved in immune defense and cell death control, and may also be hijacked by viruses to promote viral gene expression and replication [28]. The precise bidirectional role of NF-κB in BoHV-1 infection remains to be further clarified.

It is worth noting that IBRV infection typically activates multiple regulated cell death pathways in parallel rather than a single modality. Apoptosis, necroptosis and pyroptosis have all been documented to participate in IBRV pathogenesis, and these pathways share upstream signals such as ROS and NF-κB signaling with extensive crosstalk [29]. In this study, we focused on characterizing ferroptosis and its regulatory mechanism during IBRV infection, and demonstrated that inhibiting ferroptosis alone significantly suppressed viral replication, supporting its non-negligible pathogenic role. However, our data do not exclude the concurrent activation of other cell death pathways, nor do we claim ferroptosis as the dominant cell death form. The relative contribution of each pathway remains to be systematically clarified in future studies.

From a pathogenic perspective, *Bovine herpesvirus 1* is characterized by lifelong latent infection, periodic reactivation, and multiple immune evasion strategies, which underlie its persistent prevalence and intractability [30]. The ferroptosis phenotype and the *NFKB1-SLC39A8* regulatory axis identified in this study offer a new perspective for interpreting BoHV-1–host interactions: ferroptosis-mediated oxidative damage and cell death may be involved in tissue injury during viral reactivation, and NF-κB-driven iron metabolism dysregulation may also crosstalk with viral immune evasion processes. The specific roles of ferroptosis in BoHV-1 latency, reactivation and immune evasion remain to be verified in further studies.

In the context of the latest research trends in ferroptosis, this study has important insights into the cellular-level molecular mechanisms underlying IBRV-induced host cell responses, but it also has some limitations that need to be addressed in future studies. First, all experiments were conducted exclusively in vitro in MDBK cells, a classic immortalized cell line widely used for IBRV mechanism studies. Nevertheless, as IBRV naturally targets bovine respiratory epithelial cells, the physiological relevance of our findings remains limited. Validation in primary bovine respiratory epithelial cells and in vivo animal models is required to confirm the role of the *NFKB1-SLC39A8* axis in IBRV-induced ferroptosis. Second, the ferroptosis inhibitor ferrostatin-1 (Fer-1) used in this study exerts broad antioxidant and cytoprotective activities in addition to its ferroptosis-inhibitory effect; therefore, the observed reduction in viral titer upon Fer-1 treatment cannot be exclusively attributed to the blockade of ferroptosis alone. Additionally, the current conclusion regarding ferroptosis promoting viral replication is only supported by pharmacological inhibition data; we did not determine the effects of *SLC39A8* overexpression/knockdown or *NFKB1* silencing on viral replication, nor did we complement the TCID_50_ results with measurements of viral genome copies or viral protein expression. Therefore, whether the *NFKB1-SLC39A8* axis affects IBRV replication by regulating ferroptosis remains to be directly verified by genetic intervention assays in future studies. Follow-up studies using genetic interventions targeting core ferroptosis regulators (e.g., *GPX4*, *ACSL4*) are required to further confirm the independent role of ferroptosis in IBRV replication. Additional ferroptosis regulators (*PTGS2*, *LPCAT3*, *FSP1*) remain to be verified in subsequent studies. Third, although this study verifies that NFKB1 is required for *SLC39A8* upregulation and IBRV-induced ferroptosis via siRNA silencing and dual-luciferase reporter assays, we did not directly examine canonical NF-κB activation events including p65 nuclear translocation and IκB degradation. Additionally, for functional phenotypic validation in MDBK cells, only loss-of-function validation was performed for *NFKB1*; gain-of-function (overexpression) assays in MDBK cells are still lacking to confirm its sufficiency in driving *SLC39A8* expression and ferroptosis. Additionally, the dual-luciferase reporter assay only reflects the overall transcriptional activity change in the promoter and cannot confirm direct physical binding of NFKB1 to the *SLC39A8* promoter region; definitive evidence of direct transcriptional regulation requires further validation via chromatin immunoprecipitation (ChIP) assays. Therefore, the current data only support the necessity of *NFKB1* in this regulatory axis, and cannot directly confirm that the NF-κB pathway is activated upon IBRV infection. Further experiments are needed to characterize the activation dynamics and subcellular localization of NF-κB subunits during viral infection, to directly confirm that IBRV infection activates *NFKB1* and to verify the direct promoter binding of *NFKB1*. Additionally, the dual-luciferase assay was performed in HEK-293T cells; cell-type-specific transcriptional regulation may exist, and validation in MDBK cells is needed in follow-up studies. Fourth, although this study confirms that *NFKB1* functions as a transcriptional activator of *SLC39A8* during IBRV infection, it does not establish *NFKB1* as the sole regulatory transcription factor. Additional regulators including AP-1, STAT and HIF may also be involved in the transcriptional control of *SLC39A8* and collectively contribute to IBRV-induced iron metabolism dysregulation, which warrants further systematic investigation. Fifth, the specific viral factors driving *NFKB1* activation during IBRV infection, the contribution of alternative ferroptosis regulatory pathways (e.g., FSP1-CoQ10 and GCH1/BH4 pathways), and the synergistic potential of ferroptosis inhibitors with existing vaccines or antiviral agents have not been explored in the current work. Sixth, accumulating evidence has demonstrated that multiple forms of regulated cell death, including apoptosis, necroptosis and pyroptosis, are involved in IBRV pathogenesis [31]. Although we have discussed the potential coexistence and crosstalk of these pathways, the present study only focused on the characterization of ferroptosis and did not experimentally verify the activation status or relative contribution of other cell death modalities. Given that different cell death pathways often share upstream regulatory signals and interact with each other, further dissection of their synergistic or antagonistic relationships will help to fully elucidate the cell death network triggered by IBRV.

In conclusion, this study reveals that IBRV induces ferroptosis in MDBK cells via the *NFKB1-SLC39A8* axis. This finding not only provides novel insights into the pathogenic mechanism of IBRV from the perspective of regulated cell death but also identifies potential candidate molecular targets for exploring anti-IBRV interventions that antagonize viral replication. Given that the current research is limited to in vitro experimental evidence, the potential of the *NFKB1-SLC39A8* axis as a target for anti-IBRV drug development requires further in vivo validation and should be interpreted with appropriate caution. From a translational perspective, the *NFKB1-SLC39A8* axis may serve as a potential candidate target for host-directed antiviral intervention, and its practical application value for the prevention and control of IBR in cattle herds warrants further experimental validation.

## 5. Conclusions

This study demonstrates that IBRV induces ferroptosis in MDBK cells via the *NFKB1-SLC39A8* signaling axis. Mechanistically, our data support that the transcription factor NFKB1 positively regulates *SLC39A8* transcription, and *SLC39A8* functions as a key positive regulator to mediate IBRV-induced ferroptosis. Pharmacological inhibition of ferroptosis effectively reduces IBRV replication, which suggests that ferroptosis may contribute to IBRV propagation and that targeting the ferroptosis pathway may represent a promising host-directed strategy for anti-IBRV drug development.

## Figures and Tables

**Table 1:** Primer sequences used for qPCR.

Gene Name	Forward Primer (5′→3′)	Reverse Primer (5′→3′)
GPX4	GGACGACGCCCACCCTCTG	GACCATACCGCTTCACCACACAG
SLC7A11	GCACCATCATCGGAGCAGGAATC	CCAGTTCAGCGTAGGACAAGGC
ACSL4	CGTGTGGTGCTGGGACAGTTAC	TAGCCGCCTTCCTGCCAGTC
SLC39A8	ACTCAGCACCTCCATAGCCATCC	GGCTTGTCGAGTGCTCATTCCTG
FTH1	AGCTCTACGCCTCCTATGTCT	TCATCACGGTCTGGTTTCTTGAT
NFKB1	AAACACTGTGAGGATGGCGT	ATGCACCAAAAGTCCGGGAT
TFRC	GGCTCTGGCTCTCACACTCTG	TTTGCGGCTCCCTGAATAGTCC
FTL	ACCTGAATCAAGCCCTGTTGGATC	CGGAGGTTGGTCAGATGGTCAC
SLC40A1	AGGAGGAGACAGAGGCAGATTAGC	GCCGAAATGAAACCACAGCCAATG
SLC11A2	CGTCTTTGTTGTCTCGGTGTTTGC	TCCACAGCCAGTGTTGAGTTATCG
NOX4	TGGCTTTGGATTTCTGGACCTTTG	CGGATGACTTGTGACTGAGATGATG
GAPDH	TCGGAGTGAACGGATTCGG	TGATGACGAGCTTCCCGTTC

**Table 2:** siRNA sequences used in this study.

Target Gene	siRNA Name	Sense Sequence (5′→3′)	Antisense Sequence (5′→3′)
SLC39A8	siSLC39A8-879	GGCCAUUGGGACUCUAUUUTT	AAAUAGAGUCCCAAUGGCCTT
NFKB1	siNFKB1-1185	CCCAAUUUCUCGGACAGUUTT	AACUGUCCGAGAAAUUGGGTT
Negative Control	siNC	UUCUCCGAACGUGUCACGUTT	ACGUGACACGUUCGGAGAATT

## Data Availability

The original contributions presented in the study are included in the article/Supplementary Materials; further inquiries can be directed to the corresponding author.

## Supplementary Materials

The following supporting information can be downloaded at https://www.mdpi.com/article/10.3390/ani16162580/s1, Figure S1: Double restriction enzyme digestion verification of the recombinant plasmid pLenti-SLC39A8. The bovine SLC39A8 CDS fragment (1395 bp) was inserted into the pLenti-EF1a-EGFP-P2A-Puro-CMV-MCS-3Flag lentiviral vector backbone (8881 bp) for positive clone validation. M: DL10000 DNA Marker; Lane 1: Undigested empty lentiviral vector; Lane 2: Double-digested products of the recombinant overexpression plasmid, corresponding to the vector backbone (8881 bp) and the target SLC39A8 fragment (1395 bp). Data are representative of three independent experiments.

## Abbreviations

The following abbreviations are used in this manuscript:

*ACSL4*	Acyl-CoA Synthetase Long-Chain Family Member 4
*BoHV-1*	Bovine herpesvirus 1
BCA	Bicinchoninic Acid
CPE	Cytopathic Effect
DMEM	Dulbecco’s Modified Eagle Medium
DMSO	Dimethyl Sulfoxide
Fer-1	Ferrostatin-1
FBS	Fetal Bovine Serum
*FTH1*	Ferritin Heavy Chain 1
*FTL*	Ferritin Light Chain
*GPX4*	Glutathione Peroxidase 4
GSH	Glutathione
HEK-293T	Human Embryonic Kidney 293T
HRP	Horseradish Peroxidase
IBRV	Infectious Bovine Rhinotracheitis Virus
MDA	Malondialdehyde
MDBK	Madin–Darby bovine kidney
MOI	Multiplicity of infection
*NFKB1*	Nuclear Factor Kappa B1
*NOX4*	NADPH Oxidase 4
PEI	Polyethylenimine
PVDF	Polyvinylidene Fluoride
qPCR	Quantitative real-time polymerase chain reaction
ROS	Reactive Oxygen Species
SDS-PAGE	Sodium Dodecyl Sulfate Polyacrylamide Gel Electrophoresis
siRNA	Small interfering RNA
*SLC7A11*	Solute Carrier Family 7 Member 11
*SLC11A2*	Solute Carrier Family 11 Member 2
*SLC39A8*	Solute Carrier Family 39 Member 8
*SLC40A1*	Solute Carrier Family 40 Member 1 (Ferroportin)
System Xc^−^	Cystine/Glutamate Antiporter System Xc^−^
TCID_50_	50% tissue culture infectious dose
TEM	Transmission Electron Microscopy
*TFRC*	Transferrin Receptor
