# Supplementary material for: Molecular Mechanism Underlying IBRV-Induced Ferroptosis in MDBK Cells via the NFKB1-SLC39A8 Axis
Source: Animals (Basel). 2026 Aug 18;16(16):2580. doi: 10.3390/ani16162580 (PMC13508867; doi:10.3390/ani16162580)
Supplement: Supplementary file 1 [file animals-16-02580-s001.zip › animals-4438437-supplementary.pdf]

Supplementary Material

1 Supplementary Figure

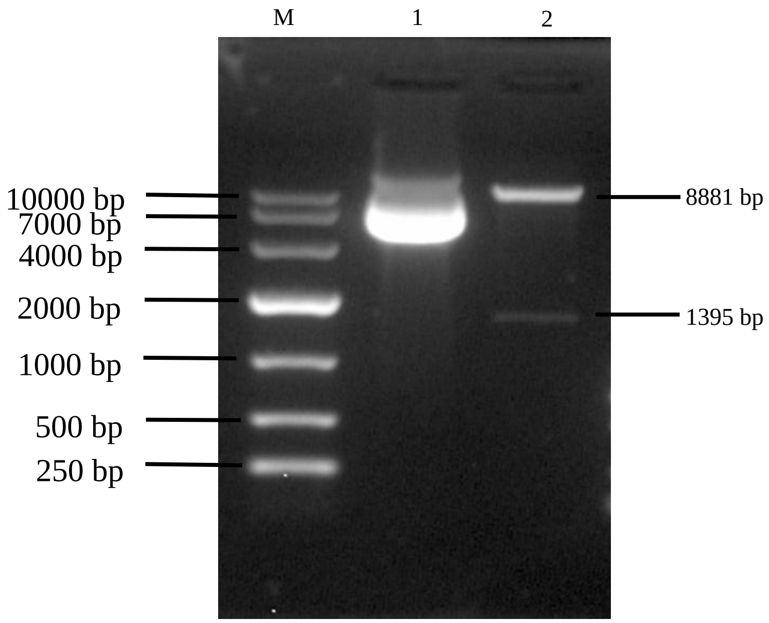

Figure S1. Double restriction enzyme digestion verification of the recombinant plasmid pLenti-SLC39A8. The bovine SLC39A8 CDS fragment (1395 bp) was inserted into the pLenti-EF1a-EGFP-P2A-Puro-CMV-MCS-3Flag lentiviral vector backbone (8881 bp) for positive clone validation. M: DL10000 DNA Marker; Lane 1: Undigested empty lentiviral vector; Lane 2: Double-digested products of the recombinant overexpression plasmid, corresponding to the vector backbone (8881 bp) and the target SLC39A8 fragment (1395 bp). Data are representative of three independent experiments.
